# The Doppelgänger phenomenon and death: a peculiar case of homicide by a subject with first-episode psychosis

**DOI:** 10.1080/20961790.2022.2055827

**Published:** 2022-06-16

**Authors:** Cristiano Barbieri, Gabriele Rocca, Caterina Bosco, Lucia Tattoli, Ignazio Grattagliano, Giancarlo Di Vella

**Affiliations:** a Section of Forensic Medicine and Forensic Sciences, University of Pavia, Pavia, Italy; b Department of Health Sciences, University of Genoa, Genoa, Italy; c S.C. Medicina Legale U, A.O.U. Città della Salute e della Scienza di Torino, Turin, Italy; d Department of Education, Psychology, Communication University Aldo Moro of Bari, Bari, Italy; e Department of Public Health and Pediatric Sciences, Legal Medicine Unit, University of Turin, Turin, Italy

**Keywords:** Forensic sciences, forensic psychiatry, homicide, first-episode psychosis, Doppelgänger, delusional disorder, criminal responsibility

## Abstract

The Doppelgänger phenomenon refers to the experience of a direct encounter with one’s self, characteriswed by: (i) the perception of a figure with one’s own identical physical features; or (ii) the apprehension that the perceived figure shares the same personality and identity. The Doppelgänger does not only look like the same person, it is his/her double. The perceptual element is usually a hallucination, although occasionally a false perception of an actual figure may be involved. This phenomenon has been described in individuals suffering from overwhelming fear, severe anxiety or intoxication, epilepsy, as well as in the sleep-wakefulness transition. It has also been reported in major psychoses. The fear of imminent death often precedes the Doppelgänger experience. This report presents the case of a 30-year-old man, Mr. Y, who was stabbed to death by Mr. X, his “double”. The aggressor and his victim, although not related, were truly doubles; remarkably, they shared the same name and surname, age, professional activity and place of work. Moreover, they attended the same sports center but barely knew each other. The forensic psychiatric evaluations in Mr. X, subsequent to the crime committed, were suggestive of a psychotic condition. This case is unique in the scientific literature. In the most serious psychotic forms, the issue of the “double” calls into question not only the dissociative processes involved in the etiopathogenesis of the disorder, but also bio-psycho-social elements, as well as personal data in this case, which made the victim and the aggressor “identical”. In the context of psychopathological functioning, the delusional mood (Wahnstimmung) that precedes the development of delirium is a sort of gateway to an impending psychotic illness, involving delusional awareness or mood (atmosphere). In psychosis, splitting is the main issue and this influence is seen as an evil, foreign, apocalyptic and unknown side no longer recognised as belonging to the self even in a physical sense. In such a situation, it is felt that the only way to survive is by suppressing one’s double as a defense against disorganisation of the self.

## Key points

People suffering from Delusional Misidentification Syndromes constitute a sub-category of psychiatric patients who may become verbally threatening and violent towards others.

“Autoscopy” is a term used for the hallucination of “seeing one’s own body at a distance” which can occur as a symptom of major psychoses such as in schizophrenia, but also in migraine and epilepsy.

Understanding the role of the “Doppelgänger phenomenon” in the progression to severely violent acts may help to elucidate factors with a potential significance in the causation of dangerous behaviours.

## Introduction

Delusional Misidentification Syndromes (DMS) are a group of phenomena whereby patients misidentify familiar persons, objects, or even themselves, believing that they have been replaced or transformed. These syndromes are delusional because the misidentifications are false and cannot be corrected in the light of experience or reason [1].

Some studies have indicated that individuals suffering from DMS constitute a sub-category of psychiatric patients who can pose significant danger to others, as they may become verbally threatening and violent as a result of their delusional misidentification [2,3].

In psychiatry, “Autoscopy” is a term used for the hallucination of “seeing one’s own body at a distance” [4]. It can occur as a symptom of major psychoses such as in schizophrenia or in migraine and epilepsy and is considered a possible explanation for the Doppelgänger phenomenon [5]. This refers to the experience of a direct encounter with one’s self, characterised by: (i) the perception of a figure with identical physical features to one’s own; or (ii) the apprehension that the figure one perceives shares one’s own personality and identity [6]. The Doppelgänger does not only look like the same person, it is his/her biologically unrelated look-alike, or double. The perceptual element is usually a hallucination, though occasionally a false perception of an actual figure may be involved [7]. This phenomenon has also been described in normal individuals suffering from overwhelming fear, severe anxiety or intoxication, as well as in the transition between sleep and wakefulness, when it may be partly a form of hypnagogic or hypnopompic hallucinations. The fear of imminent death often precedes the Doppelgänger experience [8]. The word doppelgänger is a loanword from the German, being a compound noun combining Doppel (double) and Gänger (walker or goer). Accounts of a Doppelgänger are found both in literature and in the myths and legends of a number of cultures. In Ancient Egyptian mythology, a “ka” was a concrete “spirit double” with the same memories and feelings as the counterpart person. The Doppelgänger is a version of the Ankou, a personification of death that appears in German, British and Nordic folklore. A material and spiritual interdependence between the subject and his/her double is envisaged [9].

To better understand the role of Dopplegänger phenomenon in the etiology of violence committed by mentally disordered patients, the case presented here describes a 30-year-old man, Mr. X, who stabbed to death Mr. Y, his “double”. In this very rare case, the aggressor and his victim, although they were not related and barely knew each other, remarkably shared the same name and surname, age, professional activity and place of work. The delusional misidentification was crucial in the causation of the criminal behaviour.

## Case report

One week before the homicide, the attacker, Mr. X, who had no previous history of psychiatric disturbance, had returned to live at home with his family. He appeared to them more introverted, and socially isolated in the aftermath of separation from his girlfriend, that had occurred 8 months before. One night he began to rant nonsense and to show a fixation about certain facts presented on television, rambling on about international conspiracies by the Nazis. He turned off all the lights, collapsed and then began to whisper, as if in a dissociated state, telling his mother that he was afraid of being spied on by neighbours and that the family was in danger, so he had to protect them.

The family consulted a general practitioner, who diagnosed a “depressive state” and prescribed amitriptyline and benzodiazepines for insomnia. His physical examination was unremarkable and extensive investigations, including negative EEG and CT scan. For this reason, a psychiatric visit was scheduled.

In the lead-up to the homicide, Mr. X remembered that, the day before, he felt: “*I thought I was dead, and in a suspended, timeless condition, I had heard talk about particles on television and I am made of particles so I understood that they were hunting me and wanted to carry my spirit to Hell*”.

Mr. X reported that, on the day of the homicide, “*I felt anguished and my parents advised me to go to the gym, where I saw Y, who told me “I met your sister”, that wouldn’t have been a problem but at lunch I remembered that my sister had complained of belly pain and I understood that he had raped her. I had to strike him, it was a trial of strength*”.

Therefore, the day before the appointment with the psychiatrist, the murderer went to the gym with his father. Few minutes later he crossed paths with the victim and stabbed him in the chest with a diving knife, 25 cm long. No fight was witnessed by those present at the crime scene.

When the police broke into the gym, the attacker was still inside, sitting next to a barbell and continuing to hold the bloody knife in his hand. He had a dazed expression and was looking around, appearing detached from reality. Once he had been arrested, the man showed extreme confusion and an apparent lack of awareness of what had happened.

### Forensic psychiatric assessment

In prison, at the first psychiatric evaluations, he appeared suspicious and allusive, exhibiting an initially reticent and circumspect attitude towards the specialist. At times he was disoriented, living in a delusional atmosphere in which paranoid aspects and traits of derealization and depersonalization emerged.

He maintained the considerable emotional detachment from the interlocutor. His memory seemed reduced, with signs of partial amnesia. In addition, his mimicry was sometimes incongruous, fragmentary, alternating expressions of discomfort and despair with episodes when he would burst into laughter in a totally inappropriate context. His faculty of speech was intermittent, at times slowed and at times overexcited, with episodic moments of extreme agitation.

Some days later, he appeared less suspicious but still alarmed, and easily distracted by external stimuli. He showed a depressed mood accompanied by anxiety, emotional closure and demotivation. His formal thought patterns resulted unstable, and superficial associative connections surfaced, with unstructured feelings of persecution and reference ideas. Auditory hallucinations were absent. He was affected by difficulties in falling and maintaining asleep.

His thought content showed frank mystic-philosophical delusions, and a persecution fixation permeated his contact with reality.

Among his many remarks, he said that “*a while ago a girl talked to me about a film but I couldn’t get to see it, just the trailer. This film started to insinuate ideas in my mind, making me feel anguished when I realized that I was a clone of Hitler, and that scared me*”. He was administered the WAIS-R (Wechsler Adult Intelligence Scale – Revised) which demonstrated an average intelligence, without mental deterioration. The Rorschach test was suggestive of a psychotic condition: attention and concentration skills tended to be reduced when he was emotionally involved or under stress, and progressively diminished. The examination of reality, that was only apparently adequate, highlighted overall alterations of both the perceived data and the meaning attributed to them. Adaptation to the social environment took place correctly on an objective level, but became more difficult on a subjective level, in response to an ambivalent and disharmonious affectivity that made it difficult for him to construct mature relations. The Rorschach test showed the existence of deeply rooted problems affecting the construction of the Self. His identity appeared damaged, and he experienced relations with violent and strongly persecutory connotations, also on the sexuality level.

## Discussion

At the time of the homicide, a delusional persecutory system was operating in Mr. X, in which different feelings, concerns, fears of being killed or that “something” extraordinary was about to happen, were mixed with thoughts of death. During the days before the crime, Mr. X was already experiencing, interpreting, and analysing what happened to him in a distorted and unreal sense.

A clear example of this was seen in the referred episode of “particles”.

In a climax of persecutory anguish with a psychotic matrix, his delusional experience caused a complete detachment from reality [10,11]. There was “something” against him and his family and he had to defend himself and protect his loved ones. When his “double” Mr. Y told him that he had met his sister he identified him as the enemy, by means of a clear psychotic association and concluded that “he got my sister pregnant”, because she had complained of abdominal pain.

The presence of insight into the uncanny or morbid nature of the autoscopic experience is noted in the vast majority of cases.

Some authors incorporated this into the definition of the *Doppelgänger phenomenon*. Autoscopy was defined by Faguet as a syndrome in which a person “hallucinates a vision of himself while retaining insight into the unreality of the phenomena” [12].

The Doppelgänger experience here reported cannot be encompassed within such definitions. This case argues for a less restrictive definition, such as that by Damas Mora et al. [13], who referred to “the experience of duplication of one’s real self without further qualification”.

The extraordinary of this case is that it seems between the “Doppelgänger phenomenon” by Damas Mora et al. [13] and the Capgras syndrome because the mistaken person is a real person.

This story, from a narratological point of view, recalls the problem of the splitting of the ego, well-illustrated by Dostojewsky in the novel “The double” of 1846 [14]; in fact, in the main character there is a progressive dissociation of the ego between an awkward and tormented part and another self-confident and aggressive one; and, on the psychopathological level, it is connected to the DMS: from the one of Capgras to the one of Frégoli, up to that of intermetamorphosis [15].

In a context of psychopathological functioning, the suppression of the other coincides with the defense against what has disorganized the self, and the only way to survive is by suppressing one’s double. Freud’s discovery of the unconscious made the mind an unsafe place, where the “Ego is not master in its own home” [16]. The presence of that obscure twin is perceived at the same time as unconscious, weird, even dangerous but as a product of its own mind; it is an obscure part but ego-syntonically lived [17]. In psychosis, splitting is the main issue and this influence is seen as an evil, foreign, apocalyptic and unknown side that is no longer recognised as belonging to the self even in a physical sense [18].

Jaspers [19] describes “incomprehensible experiences” of schizophrenic thought such as “the experience of feeling that the thought or action is imposed by external forces”. Influencing thought, concerns about thoughts manipulated by external engines, echoing thoughts represent some of the nuclear symptoms of schizophrenia (Schneider’s first rank symptoms) [20]. Such experiences are present in a minor form already in the premorbid period, when alterations of the boundary of self are preeminent.

The first psychiatric examination of the killer showed that he was well oriented in space, time and identity. His thought fluency was slightly slow and polarized around persecutory contents. He showed severe thought disturbances (persecutory ideas) and was extremely concerned about his neighbourhood and the neighbours intentions of spying and doing something very cruel and dangerous to his family. His mood tone was depressed and heavily worried. Memory and volition were labile and fluctuating.

Silva et al. [21] claim that the killing of the delusionally misidentified person should be viewed as a late step in a long-term process of delusional misidentification of others. Furthermore, these authors highlighted the level of fear and/or anger toward the delusionally misidentified objects as a key factor in the development of violent escalation. On the other hand, individuals may also harbour “silent” delusions and make no verbal threats before an unexpected physical attack [22].

This case report shows that the degree of threat perceived by patients as emanating from delusionally misidentified objects is the most important factor in determining the risk of committing violent acts. Impulsivity and dissociation might also play a role in the process of acting out.

The subject’s behaviour and experiences in the days before the murder (fear for the family’s safety, the feeling of being spied upon, the conviction that his sister had been raped and made pregnant, agitation and insomnia) take on an objective prodromic value in the light of the current knowledge of a predelusional state of mind, or predelusional atmosphere (*Wahnstimmung*). This expression indicates variations in the consciousness of self and in feelings about the judgment of reality that underlie the subjective and intersubjective security of the self in the world [23]. In other words, *Wahnstimmung* should be considered as the gateway to psychosis, especially of schizophrenic type, in which the predelusional mental state evokes feelings of terror and anguish produced by a progressive and indefinable transformation of the world. This leads to a perceived change in the attitudes of other people, that take on sinister, mysterious and ill-defined connotations [24,25]. There can be no doubt that all of this occurred in the case described here.

In this perspective, it is correct from the technical standpoint not only to highlight the criminogenic and criminodynamic potential of the predelusional atmosphere but also to recall the concept of delusions acting as a psychic defense mechanism [26,27]. The delusional subject operates a radical substitution of reality, that is rejected or better foreclosed (Verwerft), in the sense of expelled from the symbolic field [28]. This occurs because the intent of the delusions (especially of a persecutory nature) is to recount the primary experiences of the psychosis in an attempt to connect them and to construct a scenario that makes sense [29].

Delusions are, therefore, a defense mechanism because they attempt to reconstruct a balance that has been lost, using mechanisms that tend to incorporate the perceptive regression, the terrifying neo-reality, in a sensible account [30]. The delusional neo-reality exists because the subject is trying to recreate order in the world and succeeds to a greater or lesser extent depending on the severity of the psychotic disorder.

Mr. X killed his “alter-ego” because in his predelusional state, the man had become not only his double, but also that part of him that had raped his sister and must be eliminated.

In this state of aberrant salience the “real” similarities between the victim and perpetrator, documented in processual report, played a direct role in the attack, because amplified the psychotic atmosphere and the false conviction that Mr. X raped the sister.

## Conclusion

Understanding the role of the Doppelgänger phenomenon in the progression to severely violent acts may help to elucidate factors with a potential significance in the causation of dangerous behaviours. In particular, careful evaluation of misidentification symptoms may help to assess the risk of violence committed by psychotic individuals. Furthermore, as highlighted by Silva et al. [31], the early recognition of misidentification phenomena may be a useful indicator in managing the dangerousness of the delusional person in inpatient or correctional facilities.

In conclusion, further research on the relationship between violence and DMS is needed. Future systematic studies of large numbers of cases may improve our understanding of the psychology of aggression in psychosis.
